# Prise en charge de la radionécrose cérébrale: expérience du Service de Neurologie de l'Hôpital Militaire d'Instruction Mohammed V

**DOI:** 10.11604/pamj.2019.33.188.19325

**Published:** 2019-07-11

**Authors:** Amine Raggabi, Ahmed Bourazza, Issam Lalya

**Affiliations:** 1 Service de Neurologie, Hôpital Militaire d’Instruction Mohammed V, Rabat, Maroc; 2 Radiothérapie-oncologie, Faculté de Médecine et de Pharmacie, Université Cadi Ayyad, Marrakech, Maroc

**Keywords:** Radionécrose cérébrale, IRM, oxygénothérapie hyperbare, mesures de prévention, Cerebral radionecrosis, MRI, hyperbaric oxygen therapy, preventive measures

## Abstract

La radionécrose cérébrale est une complication rare mais redoutable de la radiothérapie externe pour les cancers ORL en particulier du nasopharynx et pour les tumeurs cérébrales. Peu de travaux se sont intéressés à cette complication aussi bien dans la population maghrébine qu'africaine. L'objectif de notre étude est donc de décrire les aspects démographiques, cliniques, paracliniques, thérapeutiques et évolutifs de la radionécrose cérébrale au sein du Service de Neurologie de l'Hôpital Militaire d'Instruction Mohammed V de Rabat (HMIMV) sur une durée de 18 ans (2000-2017). Il s'agissait de 04 femmes et 13 hommes âgés en moyenne de 50 ans. Le délai moyen entre la fin de la radiothérapie et le début des signes neurologiques était de 28 mois. La réalisation systématique d'une IRM cérébrale parfois complété d'une spectro-IRM avait permis de faire le diagnostic chez 100% des cas. Sur le plan étiologique, cette complication survenait après radiothérapie pour cancer du cavum et du larynx chez tous les cas. 15 patients ont été traités par l'association: corticothérapie en bolus, antiagrégants plaquettaires, associée à l'oxygénation hyperbare (OHB) avec une bonne évolution, et 2 patients, chez qui l'oxygénothérapie était contre-indiquée pour problème pulmonaire, et ORL, ont reçu l'association corticothérapie en bolus et antiagrégants plaquettaires avec une évolution stationnaire. À la lumière de ces résultats, nous insistons sur l'intérêt du diagnostic précoce en raison de la gravité potentielle de certains tableaux surtout neuropsychiatriques ainsi que sur le traitement par association bolus de corticoïdes + OHB car il est le meilleure garant d'une évolution favorable de ces atteintes, sans omettre le rôle crucial des mesures de prévention.

## Abstract (en)

Brain radionecrosis is a rare but life-threatening complication of external-beam radiotherapy for ENT cancers, in particular of the nasopharynx, and for brain tumors. Very few studies were conducted on this complication in the African population as well as in the Maghreb population. Therefore our study aims to describe the demographic, clinical, paraclinical, therapeutic and evolutionary features of cerebral radionecrosis in the Department of Neurology at the Mohammed V Military Teaching Hospital in Rabat over a period of 18 years (2000-2017). The study involved 4 women and 13 men, with an average age of 50 years. The mean time between the end of the radiotherapy and the onset of neurological signs was 28 months. Systematic Brain MRI, sometimes complemented by spectro-MRI, allowed the diagnosis in 100% of cases. Etiologically, this complication occurred after radiotherapy for nasopharyngeal carcinoma and cancer of the larynx in all cases. Fifteen patients were treated with a combination of: bolus application of corticosteroids, platelet aggregation inhibitors associated with hyperbaric oxygen therapy with good evolution of two patients in whom oxygen therapy was contraindicated due to a lung problem and ENT cancer, received a combination of bolus application of corticosteroids and platelet aggregation inhibitors with unchanged evolution. These results demonstrate the importance of early diagnosis in patients with potentially serious conditions, in particular neuropsychiatric conditions, as well as of treatment combining bolus application of corticosteroids and hyperbaric oxygen therapy because this is the best guarantee of a favorable outcome, without omitting the crucial role of preventive measures.

## Introduction

Aucun traitement n'est dénué de complications, et la radiothérapie comme traitement majeur du cancer n'échappe pas à cette règle. La radionécrose cérébrale est l'une des complications les plus redoutables de la radiothérapie. Le premier cas survenu après irradiation pour une néoplasie intracrânienne a été décrit par Fischer et Holfelder en 1930 [1]. La radiothérapie est considérée comme le traitement de référence dans la prise en charge des cancers de la sphère ORL surtout ceux du nasopharynx, dont la fréquence est particulièrement élevée dans notre contexte maghrébin. Cependant, elle n’est pas sans morbidité et les complications peuvent se développer à la suite de dommages aux structures voisines. Le champ de rayonnement couvre inévitablement la région médiane et inférieure des lobes temporaux du cerveau, et la base du crâne en raison de leur proximité du nasopharynx. En plus, la dose de rayonnement est habituellement de 65 à 70 Gy, qui excède la tolérance du tissu cérébral [2]. Notre travail se propose à travers une étude rétrospective ainsi qu'une revue de la littérature, de dégager les différents aspects épidémiologiques, diagnostiques ainsi que thérapeutiques et évolutifs de cette entité.

## Méthodes

Il s'agit d'étude rétrospective étalée sur 18 ans, entre 2000 et 2017 menée au sein du service de Neurologie de l'HMIMV de Rabat, portant sur 17 cas, (13 hommes et 04 femmes) d'âge moyen de 50 ans, traités pour des cancers ORL prouvés histologiquement, et atteints d'une radionécrose cérébrale. Les patients ont été traités par irradiation externe centrée sur le cavum ou le larynx et les aires ganglionnaires cervico-sus-claviculaires. La dose totale moyenne d'irradiation était de 65 Gy et le schéma de fractionnement était classique chez tous les patients (cinq fractions de 2 Gy par semaine). Dans tous les cas, les fosses infra-temporales étaient invariablement incluses dans le volume irradié. 11 patients ont reçu une chimiothérapie néo-adjuvante à base d'Adriamycine et Cisplatine à raison de trois cycles. Les examens complémentaires réalisés chez tous les patients comprenaient une IRM cérébrale parfois complétée d'une spectro-IRM. La surveillance était basée sur l'examen clinique et les examens para cliniques pour l'évaluation du contrôle tumoral et pour la détection des complications thérapeutiques. Nous avons analysé de façon rétrospective les complications neurologiques tardives survenues six mois ou plus après le début de la radiothérapie. Les cas de myélite post-radique ont été exclus de notre étude.

## Résultats

17 cas ont été considérés éligibles à notre étude, leurs caractéristiques sont présentées dans le (Tableau 1, Tableau 1(suite)).

**Tableau 1:** Résultats de l’étude

Patients	Age/sexe	Type de cancer	Radiothérapie (2 gy/fraction1j/2)	Clinique	Intervalle libre	IRM cérébrale	Traitement	Evolution
**Cas 1 2000**	46/M	Larynx	60 Gy	Syndrome alterne	10 mois	Hypersignaux cérébelleux moyen droit en T2 et FLAIR	CTC en bolus	Favorable
**Cas 2 2002**	39/M	Cavum	74 Gy	Syndrome alterne	07 mois	Hypersignaux cérébelleux moyen droit en T2 et FLAIR	CTC en bolus	Favorable
**Cas 3 2002**	41/M	Larynx	62 Gy	Syndrome alterne	08 mois	Hypersignaux cérébelleux moyen droit en T2 et FLAIR	CTC en bolus	Favorable
**Cas 4 2007**	39/M	Cavum	60 Gy	Syndrome cérébelleux latéralisé à droite	11 mois	Hypersignaux cérébelleux moyen droit en T2 et FLAIR	CTC en bolus	Stationnaire
**Cas 5 2007**	47/M	Cavum	54 Gy + chimio- thérapie	Syndrome cérébelleux	14 mois	Hypersignaux cérébelleux gauche en T2 et FLAIR	CTC + OHB	Favorable
**Cas 6 2008**	45/F	Cavum	54 Gy + chimio- thérapie	Syndrome alterne	11 mois	Hypersignaux T2 et FLAIR pons + mésencéphale	CTC en bolus	Stationnaire
**Cas 7 2010**	43/F	Cavum	54 Gy + chimio- thérapie	Syndrome alterne	14 mois	Hypersignaux cérébelleux moyen droit en T2 et FLAIR	CTC + OHB	Favorable
**Cas 8 2010**	60/M	Cavum	54 Gy + chimio- thérapie	Syndrome alterne + syndrome vestibulaire	12 mois	Hypersignaux T2 et FLAIR pons + mésencéphale	CTC + OHB	Favorable

CTC: bolus de corticoïdes, OHB : oxygénothérapie hyperbare.

**Tableau 1(suite):** Résultats de l’étude

Patients	Age/sexe	Type de cancer	Radiothérapie (2gy/fraction1j/2)	Clinique	Intervalle libre	IR M cérébrale	Traitement	Evolution
**Cas 9 2011**	67/M	Cavum	54 Gy + chimio- thérapie	Syndrome alterne	14 mois	Hypersignaux cérébelleux moyen droit en T2 et FLAIR	CTC + OHB	Favorable
**Cas 10 2011**	41/M	Cavum	54 Gy + chimio- thérapie	Syndrome alterne	16 mois	Hypersignaux cérébelleux moyens bilatéraux en T2 et FLAIR	CTC + OHB	Favorable
**Cas 11 2012**	50/F	Cavum	54 Gy + chimio- thérapie	Syndrome alterne	14 mois	Hypersignaux cérébelleux moyen gauche en T2 et FLAIR	CTC + OHB	Favorable
**Cas 12 2012**	38/M	Larynx	54 Gy + chimio- thérapie	Syndrome alterne + syndrome vestibulaire	13 mois	Hypersignaux T2 et FLAIR pons + mésencéphale	CTC + OHB	Favorable
**Cas 13 2012**	55/M	Larynx	54 Gy + chimio- thérapie	Syndrome alterne	15 mois	Hypersignaux cérébelleux moyen droit en T2 et FLAIR	CTC + OHB	Favorable
**Cas 14 2013**	70/M	Cavum	60 Gy	Syndrome vestibulaire droit + trouble cognitif	72 mois	Hypersignaux temporaux bilatéraux surtout à droite	CTC + OHB	Favorable
Cas 15 2015	42/F	Cavum	60 Gy + chimio- thérapie	HTIC + trouble cognitif	108 mois	Hypersignaux bitemporaux	CTC + OHB	Favorable
**Cas 16 2015**	53/M	Cavum	60 Gy + chimio- thérapie	Syndrome vestibulaire + trouble cognitif et syndrome pyramidal droit	96 mois	Hypersignaux temporaux gauche	CTC + OHB	Favorable
**Cas 17 2015**	74/M	Cavum	60 Gy + chimio- thérapie	Hémiparésie droite+syndrome pyramidal droit	24 mois	Hypersignaux temporaux gauche	CTB+OHB	Favorable

CTC : bolus de corticoïdes, OHB : oxygénothérapie hyperbare.

Caractéristiques démographiques: l'âge moyen des patients était de 50 ans, avec des extrêmes allant de 38 ans à 74 ans. La répartition selon le sexe trouvait une prédominance masculine avec 13 hommes et seulement 04 femmes.

Données étiologiques: les patients étaient traités par radiothérapie pour cancer du cavum chez 13 patients et pour cancer du larynx chez 04 patients. Tous les patients ont été traités par radiothérapie avec irradiation externe centrée sur le cavum ou le larynx et les aires ganglionnaires cervico-sus-claviculaires. La dose totale moyenne d'irradiation était de 65 Gy et le schéma de fractionnement était classique chez tous les patients (cinq fractions de 2 Gy par semaine). Chez 11 de ces patients un traitement par chimiothérapie néo-adjuvante à base d'Adriamycine et Cisplatine à raison de trois cycles a été instauré. Chez tous nos patients ces différentes thérapeutiques avaient permis le contrôle complet du processus tumoral.

Caractéristiques cliniques: le délai moyen entre la fin de la radiothérapie et le début des signes neurologiques était de 28 mois, comportant des extrêmes très variable avec comme délai minimal de 07 mois et maximal de 108 mois (09 ans). Les présentations cliniques comportaient de façon variable: un syndrome cérébelleux stato-cinétique latéralisé chez 03 patients. Un syndrome alterne chez 05 cas. Des troubles cognitifs (surtout mnésiques) chez 06 cas. Un syndrome d'HTIC chez 06 cas. Une symptomatologie motrice déficitaire avec irritation pyramidale chez 05 cas. Un syndrome vestibulaire central chez 02 cas. Concernant les antécédents médicaux, on trouvait surtout des facteurs de risques cardio-vasculaire à type de diabète chez 01 patient (cas 13), et à type de diabète et d'hypertension artérielle chez 03 patient (cas 9, 14, et 17).

**Figure 1:**
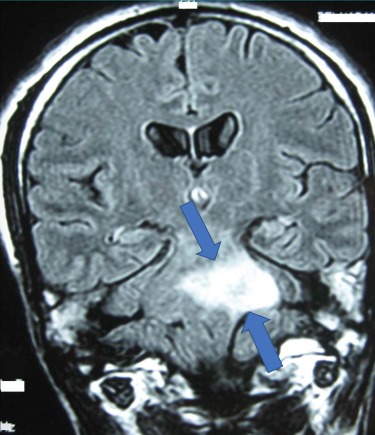
IRM cérébrale en séquence T1 avec injection de gadolinium montrant une radionécrose du tronc cérébral

**Figure 2:**
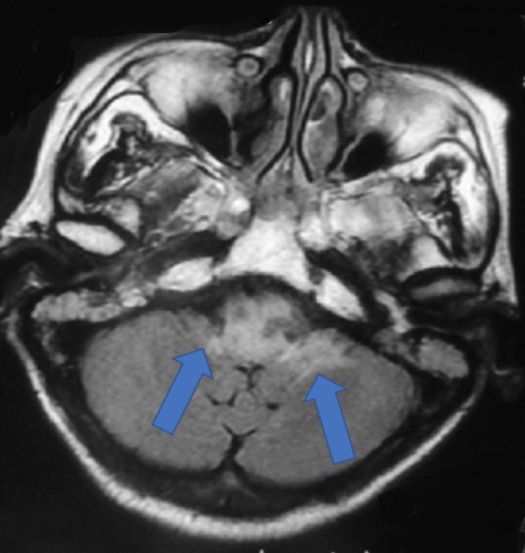
IRM cérébrale en séquence T2 avec injection de gadolinium montrant une radionécrose du tronc cérébral

**Figure 3:**
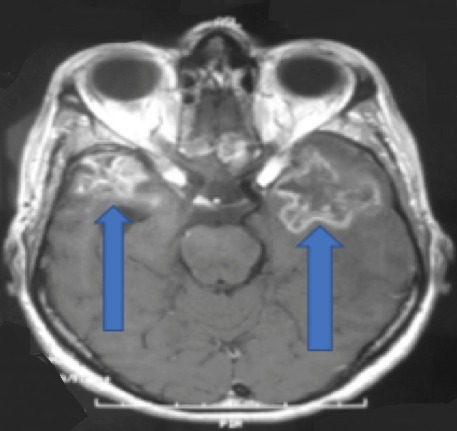
IRM cérébrale en séquence T1 montrant une radionécrose bi-temporale

Caractéristiques paracliniques: tous les patients ont bénéficié d'une IRM cérébrale comportant les séquences T1, T2, T2 FLAIR, avec injection de gadolinium, et complétée pour certains par une spectroscopie. Les complications neurologiques sont à type de nécrose cérébelleuse associée à une nécrose du tronc cérébrale dans 03 cas (Figure 1), nécrose du tronc cérébral dans 06 cas (Figure 2), et nécrose temporale dans 08 cas (Figure 3). Le bilan pré-thérapeutique pour l'OHB comprenait : une radiographie du poumon, un examen ORL et ophtalmologique, et pour le bolus de corticoïdes une bilan infectieux avec NFS et CRP.

Traitement: 15 patients ont tous été traités par l'association: corticothérapie en bolus, antiagrégants plaquettaires, associée à l'oxygénations hyperbare (10 séances en moyenne), et 2 patients, chez qui l'oxygénothérapie était contre-indiquée pour problème pulmonaire, et ORL, ont reçu l'association corticothérapie en bolus et antiagrégants plaquettaires seule.

Évolution: avec un recul moyen de 30 mois, l'évolution était marquée par une bonne réponse clinique et radiologique chez 10 patients et une stabilisation des lésions radiologiques chez 05 patients. Chez les 2 patients traités par corticothérapie en bolus et Antiagrégants seuls, avec un recul moyen de 30 mois, l'évolution était marquée par une stabilisation des signes cliniques et des lésions radiologiques sans amélioration significative.

## Discussion

**Du point de vue épidémiologique**: l'incidence de la radionécrose cérébrale varie en fonction des séries de 0.95 à 14% [1-5], avec une légère prédominance masculine. Dans notre série elle est de 10% après radiothérapie pour cancer du cavum et du larynx, ces données y compris le sex ratio concordent donc avec ceux de la littérature. Les principaux facteurs de développement d'une radionécrose cérébrale sont la dose totale, la durée de l'irradiation et surtout la dose par fraction [6-10], avec un rôle majeur de la radiothérapie hypofractionnée [3, 8] dans la survenue de cette complication. C'est ce qu'ont démontré Lee *et al.* [8], chez 1008 patients puis chez 1032 patients [3], traités par irradiation pour carcinome nasopharyngé estimant qu'un fractionnement classique (2 Gy par fraction) entrainerait un risque de nécrose de 5% à dix ans. Le rôle prépondérant du fractionnement a été également rapporté par d'autres auteurs [11]. Jen *et al.* [12], estiment ainsi que le cerveau est plus sensible au schéma hyper fractionné-accéléré avec une incidence de nécrose temporale plus élevée. Dans notre série, Tous les patients ont été traités par radiothérapie avec irradiation externe centrée sur le cavum ou le larynx et les aires ganglionnaires cervico-sus-claviculaires. La dose totale moyenne d'irradiation était de 65 Gy et le schéma de fractionnement était classique chez tous les patients (cinq fractions de 2 Gy par semaine). Les autres facteurs de risque sont l'âge avec un risque plus élevé chez les enfants et les sujets âgés, la présence de facteurs de risque vasculaires tels qu'une hypertension artérielle ou un diabète et le volume du parenchyme cérébral irradié [7]. Lee *et al*. [3] ont également montré que le stade T et la technique de radiothérapie (surtout la technique de BOOST para-pharyngé) étaient aussi des facteurs de risque. En revanche, la curiethérapie complémentaire et la chimiothérapie séquentielle n'influençaient pas significativement le risque de radionécrose temporale sauf pour la chimiothérapie concomitante à base de sel de platine qui semble augmenter le risque mais sans que cela ne soit démontré formellement [7, 9, 13]. Dans notre série, aucun patient n'a reçu un boost d'irradiation par curiethérapie, 11 patients (65%) avaient reçu une chimiothérapie néo-adjuvante à base d'Adriamycine et cisplatine. L'âge avancé (supérieur à 65 ans) est retrouvé chez seulement 03 patient (18%), ceci est probablement dû aux caractéristiques épidémiologiques des cancers oro-pharyngés dans notre contexte maghrébin touchant plus l'adulte jeune. Les facteurs de risques cardio-vasculaires sont retrouvés chez 04 patients (23,5%, avec 01 diabète et 03 diabète + HTA) ce qui concorde aussi avec la littérature.

Du point de vue clinique: sur le plan clinique, cette complication peut survenir après une période allant de six mois à 24 ans. Cependant, près de 90% des lésions deviennent symptomatiques dans les cinq ans suivant l'irradiation [6]. Cette latence reflète la capacité du parenchyme cérébral de tolérer des altérations chroniques sans élever potentiellement la pression intracrânienne [14]. Dans notre série, la période de latence moyenne était de 28 mois avec des extrêmes allant de 07 mois à 108 mois (9 ans). Cette grande variabilité de période de latence est habituelle et concorde donc avec les données de la littérature. Concernant la présentation clinique c'est un tableau de processus expansif non spécifique, avec par ordre de fréquence: crises épileptiques, pouvant être inaugurales (50%), signes d'HTIC, signes déficitaires progressifs, troubles du langage souvent d'ordre parétique, et enfin troubles cognitifs et troubles du comportement. Cheung *et al*. [15], a évalué l'impact de la radionécrose cérébrale sur la sévérité des troubles cognitifs chez 50 patients irradiés pour un carcinome nasopharyngé, il a pu démontrer une association significative entre le volume de la radionécrose et le degré des troubles cognitifs mais aussi entre le siège de la lésion cérébrale et le type de symptômes. La localisation temporale, la plus fréquemment atteinte, est associée à des troubles du langage, de la mémoire et du comportement. Dans notre série, la présentation clinique majoritaire est représentée par les le syndrome d'HTIC (06 patients) et les troubles cognitifs (06 patients), ces derniers étaient exclusivement en relation avec des localisations temporales, concordant ainsi avec l'étude menée par Cheung *et al*. La symptomatologie déficitaire essentiellement motrice se retrouve au 3^ème^ rang (05 patients), alors que dans la littérature elle est au second plan, représentant ainsi une particularité dans notre série. Une autre particularité est à signaler dans notre étude est la fréquence élevée des syndromes alterne et du syndrome cérébelleux, retrouvé respectivement chez 05 et 03 cas, en relation avec des localisations au niveau du tronc cérébrale et du cervelet. Le seul point de discorde est l'absence des crises épileptiques par rapport aux données de la littérature, du fait probablement qu'elles passent souvent inaperçues devant le cortège des autres symptômes, plus bruyants et plus durables.

**Du point de vue de l'imagerie**: sur le plan de l'imagerie, l'IRM est très sensible pour le diagnostic, avec classiquement un hyposignal en T1 et un hypersignal en T2 avec prise de contraste hétérogène et nodulaire. Wang *et al*. [4] ont montré que la nature des lésions post-radiques présente aussi certaines particularités radiologiques, avec 100% de lésions de la substance blanche dont 82% prenant le contraste et 12% de kystes post-radiques. Malgré cette sensibilité, l'IRM manque de spécificité, notamment en matière de distinction entre radionécrose et récidive tumorale [16, 17]. Cette distinction est alors facilitée par l'étude dynamique permettant d'étudier la perfusion du parenchyme cérébral, grâce à la tomographie par émission de positons (TEP) ou le SPECT [18]. Actuellement, les espoirs se portent sur la spectro-IRM avec une décroissance harmonieuse de tous les métabolites et une augmentation des lipides lors de la nécrose cellulaire [16, 17]. Enfin pour le diagnostic positif, devant l'absence de preuve anatomo-pathologique, le diagnostic repose essentiellement sur l'évolution clinique et l'aspect radiologique [19, 20]. Dans notre série, l'aspect à l'IRM cérébrale avec injection de gadolinium concordait avec les données de la littérature. L'IRM cérébrale a parfois été complétée par la spectro-IRM pour éliminer une récidive tumorale, dispensant ainsi de l'étude dynamique (PET et SPECT).

**Du point de vue thérapeutique**: concernant les aspects thérapeutiques, classiquement, 4 traitements ont été utilisés en routine pour pallier la composante nécrotique induite par la radiothérapie: l'oxygénothérapie hyperbare (OHB), les anticoagulants et antiagrégants plaquettaires, la chirurgie, et les corticoïdes. Ces traitements ont un niveau de preuve d'efficacité très différents, globalement moyen [21-24]. Les principales études, toutes rétrospectives, qui guident le choix de leur utilisation à ce jour, sont listées dans le Tableau 2. Plusieurs études récentes suggèrent un intérêt majeur du bévacizumab, un anticorps monoclonal anti-VEGF, dans le traitement des radionécroses du SNC [25-29]. Levin *et al*. [30], ont réalisé la seule étude prospective à ce jour, randomisée, en double aveugle, incluant 14 patients, comparant bévacizumab versus corticothérapie avec cross over possible où seuls les patients sous bévacizumab (12 patients au terme de l'étude) ont montré une amélioration clinique et radiologique. Aucune étude n'a été rapportée dans cette indication avec un autre anti-VEGF. Dans notre expérience, l'utilisation d'anticoagulants n'a pas été testé vu leur manque d'efficacité. Concernant l'association corticothérapie en bolus + oxygénothérapie hyperbare (10 séances), sur les 15 cas traités par cette association dans notre série, il existe une nette amélioration radiologique, toujours suivie d'une stabilisation, voire d'une amélioration clinique. La dose du traitement par corticoïdes est alors diminuée pour réduire le risque d'effets indésirables. Ces résultats semblent meilleurs que ceux de la littérature ce qui représente une particularité intéressante de notre étude. Chez 2 patients de notre série chez qui l'utilisation d'oxygénothérapie hyperbare était contre-indiquée (pour problèmes ORL et pulmonaire), l'usage seul de corticoïdes en bolus associée à un traitement antiagrégant a permis seulement de stabiliser les lésions radiologiques et l'état clinique, sans toutefois permettre une amélioration. Soulignons aussi le fait que aucun patient de notre série n'a bénéficié d'un traitement par bévacizumab, vu sa non disponibilité au moment de l'étude.

**Tableau 2:** Principales études sur les thérapeutiques utilisées pour la radionécrose cérébrale

Étude	traitement	année	Patient (n)	Type de tumeur	Latence des symptomes (mois)	Amélioration clinique	Conclusion ou commentaires
Chuba et al [21]	OHB	1997	10	cérébrale	7,6	10	-Biais d’interprétation Amélioration nette chez 2 patients; absence d’informations précises pour les autres -Traitement concomitant par corticoïdes
Leber et al [22]	OHB	1998	2	MAV	-	1	Amélioration en l’absence de traitement par corticoïdes
Glantz et al [23]	ATC	1994	11	Cérébrale (8) ADK côlon, membre supérieur, cæcum	24	8	Étude ouverte mais efficacité clinique très modérée
Happold et al [24]	ATC	2008	8	Cérébrales (3) Myélome multiple (2) ORL (1) Lymphome périphérique (1) Sein (1)	3	-	Traitement pas dangereux mais efficacité très modeste (conclusion des auteurs)

ATC : anticoagulant, OHB : oxygénothérapie hyperbare, MAV : malformation artério-veineuse

**Du point de vue des mesures de prévention**: en dernier lieu, la prévention demeure l'un des aspects le plus important. Un cache du parenchyme cérébral est l'une des mesures efficaces, mais parfois difficile du fait de la proximité de la partie inférieure des lobes temporaux qui sont invariablement inclus dans le volume irradié. Pour la dose totale, elle ne peut être diminuée du fait de son importance dans le contrôle local des carcinomes nasopharyngés, par contre l'utilisation d'une dose par fraction ne dépassant pas 2 Gy permet d'améliorer la tolérance des tissus sains avoisinants comme l'ont démontré plusieurs auteurs et donc de minimiser le risque de radionécrose cérébrale [3, 8, 12]. Enfin, l'utilisation des nouvelles techniques d'irradiation comme la radiothérapie conformationnelle avec modulation d'intensité, et la protonthérapie, permettent d'administrer de fortes doses à la tumeur en protégeant les organes à risque et de ce fait diminuer le risque de radionécrose cérébrale. Ceci concorde avec notre étude puisque l'incidence a diminué après 2011 (02 cas, irradiés entre 2012 et 2017) après l'acquisition par le service de radiothérapie de notre hôpital de l'arc thérapie dynamique volumétrique (RapidArc), nouvelle technique moins pourvoyeuse de complications post-radiques.

## Conclusion

La radiothérapie est un traitement très efficace dans la prise en charge des tumeurs du système nerveux central (SNC) et des tumeurs de la base du crâne. La radionécrose cérébrale focale, est une complication tardive mais redoutable et potentiellement invalidante de la radiothérapie externe. Son incidence est probablement sous-estimée, à la fois chez les patients traités pour une tumeur cérébrale et surtout chez les patients irradiés pour des tumeurs extra neurologiques telles que les tumeurs du nasopharynx comme ce fut le cas dans notre série. Traditionnellement, le traitement utilisé dans le cadre des radionécroses du SNC repose sur les corticoïdes, avec un effet transitoire. Des études ouvertes ont testé divers traitements chez l'homme (agents anti thrombotiques, oxygénothérapie en caisson hyperbare, chirurgie, vitamine E), mais aucune ne rapporte un niveau de preuve d'efficacité suffisant. Toutefois, notre étude montre une bonne réponse thérapeutique pour l'association corticothérapie et oxygénothérapie hyperbare. Il existe aujourd'hui un intérêt particulier pour l'utilisation du bévacizumab dans cette indication. Enfin, une meilleure connaissance de la physiopathologie devrait permettre à l'avenir l'utilisation de thérapies mieux ciblées.

### État des connaissances actuelles sur le sujet

- La radionécrose cérébrale est une situation assez fréquente et dont les étiologies sont dominées par la radiothérapie pour tumeurs cérébrales et pour tumeurs naso-pharayngées chez des sujets âgés;
- Habituellement révélée par des crises épileptiques, des signes d'HTIC avec troubles cognitifs, et par des signes déficitaires;
- Différentes thérapeutiques ont été utilisées dans le traitement de la radionécrose cérébrale, mais aucune n'a fait preuve d'efficacité suffisante, excepté récemment pour le Bévacizumab.

### Contribution de notre étude à la connaissance

- Cette complication survient exclusivement après radiothérapie pour cancers naso-pharyngés et chez une population plus jeune, du fait des caractéristiques épidémiologique des cancers naso-pharyngés dans notre contexte maghrébin;
- La symptomatologie est assez polymorphe, avec peu de crises épileptiques, mais plutôt avec une proportion assez importante de syndrome alterne;
- L'association oxygénothérapie hyperbare et corticoïdes semble plutôt efficace pour le traitement de la radionécrose cérébrale, il est à souligner également l'importance des mesures de prévention surtout l'utilisation des nouvelles techniques de radiothérapie qui réduisent considérablement l'incidence de cette complication.

## Conflits des intérêts

Les auteurs ne déclarent aucun conflit d'intérêts.

## Remerciements

Nos sincères remerciements à tout le staff de l'Université Mohammed V Souissi et Cadi Ayyad de Marrakech.
